# Chemical Composition and Immunomodulatory Activity of *Hypericum perforatum* Essential Oils †

**DOI:** 10.3390/biom10060916

**Published:** 2020-06-17

**Authors:** Igor A. Schepetkin, Gulmira Özek, Temel Özek, Liliya N. Kirpotina, Andrei I. Khlebnikov, Mark T. Quinn

**Affiliations:** 1 Department of Microbiology and Immunology, Montana State University, Bozeman, MT 59717, USA; igor@montana.edu (I.A.S.); liliya.kirpotina@montana.edu (L.N.K.); 2 Department of Pharmacognosy, Faculty of Pharmacy, Anadolu University, Eskişehir 26470, Turkey; gulmiraozek@gmail.com (G.Ö.); temelozek@gmail.com (T.Ö.); 3 Medicinal Plant, Drug and Scientific Research and Application Center (AUBIBAM), Anadolu University, Eskişehir 26470, Turkey; 4 Kizhner Research Center, Tomsk Polytechnic University, Tomsk 634050, Russia; aikhl@chem.org.ru; 5 Scientific Research Institute of Biological Medicine, Altai State University, Barnaul 656049, Russia

**Keywords:** *Hypericum perforatum*, essential oil, calcium flux, neutrophil, chemotaxis, reactive oxygen species, sesquiterpene, anti-inflammatory

## Abstract

*Hypericum* L. (Hypericaceae) extracts have been used for their therapeutic effects; however, not much is known about the immunomodulatory activity of essential oils extracted from this plant. We isolated essential oils from the flowers and leaves of *H. perforatum* and analyzed their chemical composition and innate immunomodulatory activity. Analysis of flower (HEO_Fl_) versus leaf (HEO_Lv_) essential oils using gas chromatography–mass spectrometry revealed that HEO_Fl_ was comprised mainly of monoterpenes (52.8%), with an abundance of oxygenated monoterpenes, including *cis*-*p*-menth-3-en-1,2-diol (9.1%), α-terpineol (6.1%), terpinen-4-ol (7.4%), and limonen-4-ol (3.2%), whereas the sesquiterpenes were found in trace amounts. In contrast, HEO_Lv_ was primarily composed of sesquiterpenes (63.2%), including germacrene D (25.7%) and β-caryophyllene (9.5%). HEO_Lv_ also contained oxygenated monoterpenes, including terpinen-4-ol (2.6%), while monoterpene hydrocarbons were found in trace amounts. Both HEO_Fl_ and HEO_Lv_ inhibited neutrophil Ca^2+^ mobilization, chemotaxis, and reactive oxygen species (ROS) production, with HEO_Lv_ being much more active than HEO_Fl_. Furthermore, the pure sesquiterpenes germacrene D, β-caryophyllene, and α-humulene also inhibited these neutrophil responses, suggesting that these compounds represented the active components of HEO_Lv_. Although reverse pharmacophore mapping suggested that potential protein targets of germacrene D, β-caryophyllene, bicyclogermacrene, and α-humulene could be PIM1 and mitogen-activated protein kinase (MAPK)-activated protein kinase 2 (MAPKAK2), a kinase binding affinity assay did not support this finding, implying that other biological targets are involved. Our results provide a cellular and molecular basis to explain at least part of the beneficial immunotherapeutic properties of the *H. perforatum* essential oils.

## 1. Introduction

*Hypericum* L. (Hypericaceae) is comprised of approximately 500 species that can be found around the world. Various products from *Hypericum* species have been used as antidepressant, sedative, diuretic, antiphlogistic, analgesic, astringent, and antipyretic remedies in Europe, America, Africa, and Asia [1,2,3,4,5,6,7,8]. One of the most intensively studied medicinal plants from this genus is *H. perforatum* L. or St. John’s wort, which is a perennial herb that his known for its beneficial pharmacological properties [2,3]. For example, *H. perforatum* L. has been widely used in many countries in antibacterial, antiviral, anti-inflammatory, antinociceptive, or analgesic remedies [4]. Extracts from this herb have also been reported as a therapeutic remedy for burns, skin wounds, cuts, stomach aches, and ulcers [5]. In addition, *H. perforatum* extracts have also been reported to have anti-angiogenic, anti-fibroblastic, and antioxidant properties [6,7,8]. The phytochemical profile of *H. perforatum* includes naphthodianthrones (specifically hypericin and pseudohypericin), hyperforin, proanthocyanins, flavonoids, biflavonoids, xanthones, phenylpropanes, phenolic acids, and volatile constituents [9,10,11]. *Hypericum* essential oils are rich sources of monoterpenes, sesquiterpenes, and their oxygenated derivatives (reviewed in [9] and Table 1, which has a listing of the more recent *H. perforatum* essential oil data published after this review).

Essential oils are natural mixtures of terpenes, which have a wide range of pharmacological activities [12]. The chemical composition and biological activity of essential oils can be affected by many factors, including harvesting time and which part of the plant is used for essential oil isolation [13]. Essential oils prepared from various plant species have become increasingly popular in recent decades as complementary and alternative medicines. Thus, analysis of the chemical composition of essential oils from different plant species and subsequent evaluation their biological properties, including immunomodulatory activity, can lead to the discovery of novel immunomodulatory agents that may be useful for therapeutic purposes. Although previous studies have demonstrated that *Hypericum* essential oils have antimicrobial, anti-proliferative, and antioxidant activities [14,15,16,17,18], the innate immunomodulatory effects of *Hypericum* essential oils have not been investigated.

The innate immune system is essential for host defense and provides immediate defense against infection. Among the earliest cell types responding to invasion by pathogens are innate immune cells, such as neutrophils and monocyte/macrophages [19]. Neutrophils perform a variety of microbicidal functions, including phagocytosis, chemotaxis, and biochemical destruction of pathogens [20]. Thus, neutrophils represent an ideal pharmacological target for therapeutic development, and a number of small molecules that modulate neutrophil function have been identified [21,22,23]. In addition, numerous natural products, including essential oils, have been evaluated for immunomodulatory activity. For example, we recently analyzed the chemical composition of essential oils from *Artemisia kotuchovii* Kupr, *Ferula akitschkensis* B.Fedtsch. ex Koso-Pol., and *Ferula iliensis* Krasn. ex Korovin and characterized their neutrophil modulatory activity [24,25,26]. 

Based on the reported therapeutic effects of *H. perforatum* extracts, we hypothesized that *H. perforatum* essential oils might have immunomodulatory activity. Thus, we evaluated the chemical composition and neutrophil immunomodulatory activity of essential oils obtained from flowers and leaves of *H. perforatum*.

## 2. Materials and Methods

### 2.1. Plant Material

*Hypericum perforatum* was collected in 2019 during the flowering and fruiting stages on the south side of Baldy Mountain, Gallatin Valley, Montana, USA (45.7674° N, 110.9438° W) at an elevation of ~1800 m above sea level. Flowers and leaves were air-dried for 7–10 days at room temperature in the dark before hydrodistillation. Botanical identification of the plant material was performed by botanist Robyn A. Klein from Montana State University (Bozeman, MT, USA).

### 2.2. Materials

Dimethyl sulfoxide (DMSO), *N*-formyl-Met-Leu-Phe (*f*MLF), phorbol 12-myristate 13-acetate (PMA), Histopaque 1077, α-terpineol, myrtenol, and γ-terpinene were purchased from Sigma-Aldrich Chemical Co. (St. Louis, MO, USA). Geraniol, germacrene D, α-humulene, and β-caryophyllene were from Cayman Chemicals (Ann Arbor, MI, USA), and (-)-terpinen-4-ol was purchased from Tokyo Chemical Industry Co. (Tokyo, Japan). *n*-Hexane was purchased from Merck (Darmstadt, Germany). Fluo-4AM was purchased from Invitrogen (Carlsbad, CA, USA). L-012 was purchased from Tocris Bioscience (San Diego, CA, USA). Hanks’ balanced salt solution (HBSS; 0.137 M NaCl, 5.4 mM KCl, 0.25 mM Na_2_HPO_4_, 0.44 mM KH_2_PO_4_, 4.2 mM NaHCO_3_, 5.56 mM glucose, and 10 mM HEPES, pH 7.4) was purchased from Life Technologies (Grand Island, NY, USA). HBSS without Ca^2+^ and Mg^2+^ is designated as HBSS^−^; HBSS containing 1.3 mM CaCl_2_ and 1.0 mM MgSO_4_ is designated as HBSS^+^.

### 2.3. Essential Oil Extraction

Essential oils were obtained by hydrodistillation of dried plant material using a Clevenger apparatus, as previously described [26]. We used conditions accepted by the European Pharmacopoeia (European Directorate for the Quality of Medicines, Council of Europe, Strasbourg, France, 2014) to avoid artifacts. The yield of the essential oil was calculated based on the amount of air-dried plant material used. Stock solutions of the essential oils were prepared in DMSO (10 mg/mL) for biological evaluation and in *n*-hexane (10% *w*/*v*) for gas-chromatographic analysis.

### 2.4. Gas Chromatography–Mass Spectrometry (GC-MS) Analysis

GC-MS analysis was performed using an Agilent 5975 GC-MSD system (Agilent Technologies, Santa Clara, CA, USA), as reported previously [27]. An Agilent Innowax FSC column (60 m × 0.25 mm, 0.25 μm film thickness) was used with He as the carrier gas (0.8 mL/min). The GC oven temperature was kept at 60 °C for 10 min, increased to 220 °C at a rate of 4 °C/min, kept constant at 220 °C for 10 min, and then increased to 240 °C at a rate of 1 °C/min. The split ratio was adjusted to 40:1, and the injector temperature was 250 °C. MS spectra were monitored at 70 eV with a mass range of 35 to 450 m/z.

GC analysis was performed on an Agilent 6890N GC system. To obtain the same elution order as with GC-MS, the line was split for FID and MS detectors, and a single injection was performed using the same column and appropriate operational conditions. The ionization detector (FID) temperature was 300 °C. Essential oil components were identified by co-injection with standards (whenever possible), which were purchased commercially or isolated from natural sources. In addition, compound identities were confirmed by comparison of their mass spectra with those in the Wiley GC/MS Library (Wiley, NY, USA), MassFinder software 4.0 (Dr. Hochmuth Scientific Consulting, Hamburg, Germany), Adams Library, and NIST Library. Confirmation was also achieved using the in-house “Başer Library of Essential Oil Constituents” database, obtained from chromatographic runs of pure compounds performed with the same equipment and conditions. A C_8_–C_40_ *n*-alkane standard solution (Fluka, Buchs, Switzerland) was used to spike the samples for the determination of relative retention indices (RRI). Relative percentage amounts of the separated compounds were calculated from FID chromatograms.

### 2.5. Isolation of Human Neutrophils

Neutrophils were isolated from blood that was collected from healthy donors in accordance with a protocol approved by the Institutional Review Board at Montana State University (Protocol #MQ041017). Neutrophils were purified from the blood using dextran sedimentation, followed by Histopaque 1077 gradient separation and hypotonic lysis of red blood cells, as described previously [22]. Isolated neutrophils were washed twice and resuspended in HBSS^−^. Neutrophil preparations were routinely >95% pure, as determined by light microscopy, and >98% viable, as determined by trypan blue exclusion. Neutrophils were obtained from multiple different donors (n = 8); however, the cells from different donors were never pooled during experiments.

### 2.6. Ca^2+^ Mobilization Assay

Changes in neutrophil intracellular Ca^2+^ concentrations ([Ca^2+^]_i_) were measured using a FlexStation 3 scanning fluorometer (Molecular Devices, Sunnyvale, CA, USA). Briefly, human neutrophils suspended in HBSS^−^ were loaded with Fluo-4AM at a final concentration of 1.25 μg/mL and incubated for 30 min in the dark at 37 °C. The cells were then washed with HBSS^−^, resuspended in HBSS^+^, and aliquoted into the wells of flat-bottom, half-area 96-well black microtiter plates (2 × 10^5^ cells/well). Essential oils or pure compounds diluted in DMSO were added to the wells (final concentration of DMSO was 1%). The samples were preincubated for 10 min, followed by addition of 5 nM *f*MLF. Changes in fluorescence were monitored (λ_ex_ = 485 nm, λ_em_ = 538 nm) every 5 s for 240 s at room temperature after addition of the test compound/oil. The maximum change in fluorescence, expressed in arbitrary units over baseline, was used to determine the response. Responses were normalized to the response induced by 5 nM *f*MLF, which was assigned a value of 100%. Curve fitting (at least five or six points) and calculation of median effective concentration values (EC_50_ or IC_50_) were performed by nonlinear regression analysis of the dose–response curves generated using Prism 7 (GraphPad Software, Inc., San Diego, CA, USA). 

### 2.7. Chemotaxis Assay

Human neutrophils were resuspended in HBSS^+^ containing 2% (*v*/*v*) heat-inactivated fetal bovine serum (2 × 10^6^ cells/mL), and chemotaxis was analyzed in 96-well ChemoTx chemotaxis chambers (Neuroprobe, Gaithersburg, MD). After preincubation with the indicated concentrations of the test sample (essential oil or pure compound) or DMSO (1% final concentration) for 30 min at room temperature, the cells were added to the upper wells of the ChemoTx chemotaxis chambers. The lower wells were loaded with 30 µL of HBSS^+^ containing 2% (*v*/*v*) fetal bovine serum and the indicated concentrations of test sample, DMSO (negative control), or 1 nM *f*MLF as a positive control. Neutrophils were allowed to migrate through the 5.0-µm pore polycarbonate membrane filter for 60 min at 37 °C and 5% CO_2_. The number of migrated cells was determined by measuring ATP in lysates of transmigrated cells using a luminescence-based assay (CellTiter-Glo; Promega, Madison, WI, USA), and luminescence measurements were converted to absolute cell numbers by comparison of the values with standard curves obtained with known numbers of neutrophils. Curve fitting (at least eight to nine points) and calculation of median effective concentration values (IC_50_) were performed by nonlinear regression analysis of the dose–response curves generated using GraphPad Prism 8.

### 2.8. ROS Production Assay

ROS production was determined by monitoring L-012-enhanced chemiluminescence, which is a reliable method for detecting superoxide anion (O_2_^−^) production [22]. Human neutrophils were resuspended at 2 × 10^5^ cells/mL in HBSS^+^ supplemented with 40 µM L-012. Cells (100 µL) were aliquoted into wells of 96-well flat-bottomed microtiter plates containing essential oil or compounds at different concentrations (final DMSO concentration of 1%). Cells were preincubated for 10 min, and 200 nM PMA was added to each well to stimulate ROS production. Luminescence was monitored for 120 min (2-min intervals) at 37 °C using a Fluoroskan Ascent FL microtiter plate reader (Thermo Electron, Waltham, MA, USA). The curve of light intensity (in relative luminescence units) was plotted against time, and the area under the curve was calculated as total luminescence. Compound concentrations that inhibited ROS production by 50% of the PMA-induced response (positive control) were determined by graphing the percentage inhibition of ROS production versus the logarithm of concentration of test sample (IC_50_). Each curve was determined using five to seven concentrations.

### 2.9. Kinase K_d_ Determination

Selected sesquiterpenes were submitted for dissociation constant (K_d_) determination toward PIM1 and MAPKAPK2 using KINOMEscan [28] (Eurofins Pharma Discovery, San Diego, CA, USA). For dissociation constant K_d_ determination, a 12-point half-log dilution series (a maximum concentration of 33 µM) was used. Assays were performed in duplicate, and their average mean value is displayed.

### 2.10. Human Neutrophil Elastase (HNE) Inhibition Assay

Essential oils and individual compounds were dissolved in 100% DMSO at 5 mM stock concentrations. The final concentration of DMSO in the reactions was 1%, and this level of DMSO had no effect on enzyme activity. Sivelestat, a known HNE inhibitor, was used as a positive control. The inhibition assay was performed, as described previously [29]. Briefly, a solution containing 200 mM Tris-HCl (pH 7.5), 0.01% bovine serum albumin, 0.05% Tween-20, and 20 mU/mL of human neutrophil elastase was added to black, flat-bottom 96-well microtiter plates containing different concentrations of test compounds. Reactions were initiated by addition of 25 µM elastase substrate *N*-methylsuccinyl-Ala-Ala-Pro-Val-7-amino-4-methylcoumarin in a final reaction volume of 100 µL/well. Kinetic measurements were obtained every 30 s for 10 min at 25 °C using a Fluoroskan Ascent FL fluorescence microplate reader (Thermo Electron, MA, USA) with excitation and emission wavelengths at 355 and 460 nm, respectively. The concentration of compound that caused 50% inhibition of the enzymatic reaction (IC_50_) was calculated by plotting % inhibition versus logarithm of inhibitor concentration.

### 2.11. Cytotoxicity Assay

Human promyelocytic leukemia HL-60 cells were cultured in RPMI-1640 medium supplemented with 10% heat-inactivated FBS, 10 mM HEPES, 100 µg/mL streptomycin, and 100 U/mL penicillin. Cytotoxicity was analyzed with a CellTiter-Glo Luminescent Cell Viability Assay Kit (Promega, Madison, WI, USA), according to the manufacturer’s protocol. Briefly, HL-60 cells were cultured at a density of 1 × 10^5^ cells/well with different concentrations of essential oil or compound (final concentration of DMSO was 1%) for 30 min or 2 h at 37 °C and 5% CO_2_. Following treatment, substrate was added to the cells, and the samples were analyzed with a Fluoroskan Ascent FL microplate reader.

### 2.12. Molecular Modeling

**Figure 1:**
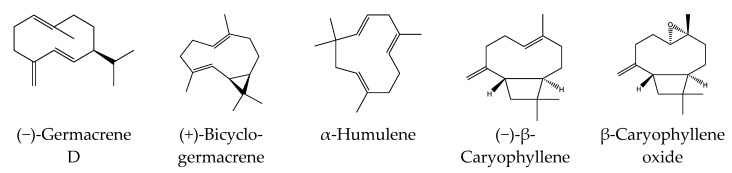
Chemical structures of sesquiterpenes (−)-germacrene D, (+)-bicyclogermacrene, α-humulene, (−)-β-caryophyllene, and β-caryophyllene oxide.

Structures of the main sesquiterpenes found in HEO_Lv_ and used for molecular modeling are shown in Figure 1. The protein targets for β-caryophyllene, (−)-germacrene D, (+)-bicyclogermacrene, and α-humulene were analyzed using the PharmMapper Server [30]. This online tool is intended to recognize potential target possibilities for a given small molecule through an “invert” pharmacophore mapping approach. The software uses several built-in reference databases of protein drug targets encoded by sets of pharmacophore points for faster mapping. Initial 3D structures of the investigated compounds were downloaded from the PubChem database (https://pubchem.ncbi.nlm.nih.gov) and saved in Tripos MOL2 format. The MOL2 files of (−)-β-caryophyllene, (−)-germacrene D, (+)-bicyclogermacrene, and α-humulene (PubChem compound CIDs: 5281515, 5317570, 5315347, and 5281520, respectively; see Figure 1) were uploaded into the PharmMapper web server. Automatic generation of up to 300 conformers for each compound was switched on. The “Human Protein Targets Only” database containing 2241 targets was selected for pharmacophore mapping. The top 250 potential targets were retrieved and sorted by normalized fit score value. The physicochemical properties of selected compounds were computed using SwissADME (http://www.swissadme.ch) [31]. 

## 3. Results and Discussion

### 3.1. Essential Oil Composition

Although the chemical composition of *H. perforatum* essential oils has been reported previously in several publications [9,11,32,33,34,35,36,37,38,39], there is a wide variation in the reported levels of secondary metabolites from different *H. perforatum* plant samples (see Table 1 for a summary of results from recent studies since 2010). This variability can impact the specific pharmacological activity of essential oils/extracts [40,41]. In addition, few studies have investigated flower and leaf essential oils separately, and there are no publications on the chemical composition of essential oils from *H. perforatum* collected in the Rocky Mountain region of the United States. Thus, we analyzed the essential oil composition of flowers and leaves from *H. perforatum* samples collected in this region.

The extraction yields (*v*/*w*) of essential oils obtained from *H. perforatum* flowers (designated as HEO_Fl_) and leaves (HEO_Lv_) were 0.3% (HEO_Fl_) and 0.3% (HEO_Lv_). The chemical composition of the oils was evaluated using GC-FID and GC/MS simultaneously, and Table 2 and Table 3 summarize the identified compounds, their percentage composition, and their relative retention indices (RRI) (compounds are listed in order of their elution). A total of 94 constituent compounds were identified in the *H. perforatum* essential oils. Thirty compounds were identified in HEO_Fl_, representing around 71.3% of the total essential oil composition. The main components of HEO_Fl_ were 3-methoxy-2,3-dimethylcyclobutene (9.8%), *cis*-*p*-menth-3-en-1,2-diol (9.1%), terpinen-4-ol (7.4%), α-terpineol (6.1%), *trans*-ascaridol glycol (4.6%), 4-hydroxy-4-methyl-cyclohex-2-enone (3.4%), limonen-4-ol (3.2%), *p*-cymen-8-ol (2.9%), myrtenol (2.7%), and α-pinene (2.2%). Twenty other compounds were present at concentrations <2.0%. Seventy-five compounds were identified in HEO_Lv_, representing around 97.2% of the total essential oil composition. The main components of HEO_Lv_ were germacrene D (25.7%), β-caryophyllene (9.5%), terpinen-4-ol (7.4%), sabinene (5.6%), α-pinene (4.9%), β-pinene (4.6%), (*E*)-β-ocimene (4.2%), bicyclogermacrene (2.5%), δ-cadinene (2.5%), and myrcene (2.1%). Fifty-five other compounds were present at concentrations from 0.1% to <2%. The remaining 10 volatile compounds were identified in trace amounts (<0.1%). Overall, there were significant differences in essential oil composition between *H. perforatum* flowers and leaves, with the major components of flowers being oxygenated monoterpenes (49.2%) and the main components of the leaves being sesquiterpene hydrocarbons (52.9%), including very high levels of germacrene D (25.7%).

The chemical composition of *H. perforatum* essential oils obtained from aerial parts of the plant has been shown previously to vary according to the collection period and location of the plants collected [44]. Comparison of the chemical profiles of HEO_Fl_ and HEO_Lv_ with those reported previously from other locations showed that they all contained common sesquiterpene constituents, such as β-caryophyllene and germacrene D, and indicated similarities with *H. perforatum* essential oils from Lithuania [45]. For example, the concentrations of β-caryophyllene and caryophyllene oxide in essential oils from leaves were higher than those from flowers, whereas dodecanol, spathulenol, viridiflorol, carotol, and tetradecanol were present in higher quantities in flowers from *H. perforatum* collected in Lithuania [45,46]. Based on these compounds, *H. perforatum* essential oils from Lithuania were classified into three chemotypes: β-caryophyllene, caryophyllene oxide, and germacrene D [45,46]. Similarly, analysis of the chemical composition of the essential oils from flower, leaf, and stems of *H. perforatum* collected in Serbia revealed that the highest concentration of non-terpene compounds was found in the flower and stem essential oils, while high concentrations of sesquiterpenes were characteristic of leaf essential oils [44]. Finally, essential oils isolated from *H. perforatum* collected in Uzbekistan have been reported to contain β-caryophyllene as their main constituent [47].

### 3.2. Effect of the Essential Oils and Their Components on Neutrophil Functional Responses

Essential oils and their components have been reported previously to modulate intracellular Ca^2+^ flux and inhibit cell migration [24,25,26]. We screened *Hypericum* essential oils for neutrophil immunomodulatory activity and evaluated the effects of HEO_Fl_ and HEO_Lv_ and selected compounds on neutrophil activation.

**Figure 2:**
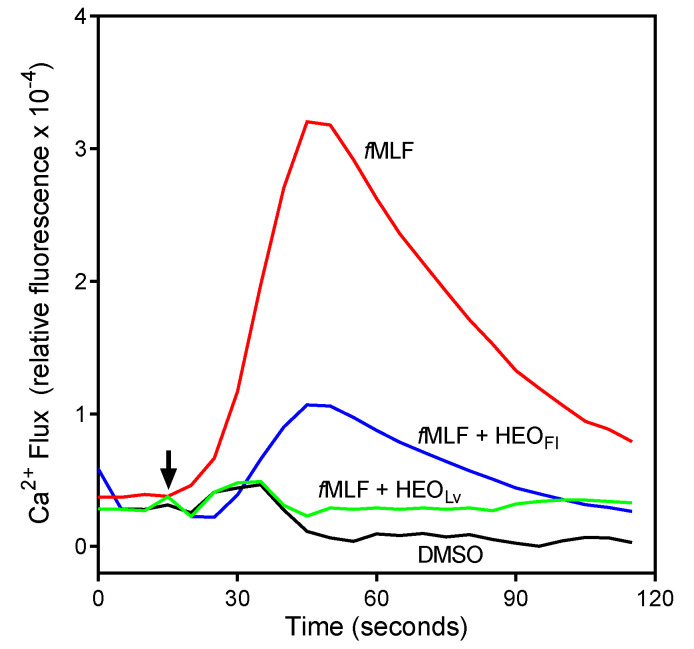
Effect of HEO_Lv_ and HEO_Fl_ on *f*MLF-induced Ca^2+^ mobilization in human neutrophils. Human neutrophils were pretreated for 10 min with 25 µg/mL of the indicated essential oil or 1% DMSO (negative control), followed by stimulation with 5 nM *f*MLF (indicated by arrow), and Ca^2+^ flux was monitored for the indicated times. The data are from one experiment that is representative of three independent experiments.

As shown in Table 4, both HEO_Fl_ and HEO_Lv_ inhibited intracellular Ca^2+^ flux in *f*MLF activated neutrophils, although HEO_Lv_ was ~5-fold more potent than HEO_Fl_. A representative time course for the inhibition of *f*MLF-stimulated Ca^2+^ flux by HEO_Fl_ and HEO_Lv_ (25 µg/mL each) is shown in Figure 2. We next considered the effects of individual constituent compounds on neutrophil Ca^2+^ mobilization in an effort to identify the active component(s). Previously, we analyzed the effect of a number of these same compounds on neutrophil Ca^2+^ flux, including 16 compounds that we found here to comprise 24.0% of HEO_Fl_ and 29.6% of HEO_Lv_ [24,26]. These data are included in Table 4 for reference. As shown in Table 4, β-pinene, sabinene, and γ-terpinene, which represent 11.3% of HEO_Lv_, were found previously to have no effect on neutrophil Ca^2+^ mobilization and thus are likely not involved in the inhibitory effects of *Hypericum* essential oils. In contrast, we found previously that 6-methyl-3,5-heptadien-2-one (MHDO) inhibited neutrophil Ca^2+^ flux, although it is present in only trace amounts in HEO_Fl_ (<1.0%). Thus, it is possible that MHDO contributes to the inhibition observed with HEO_Fl_ treatment, but it is more likely that there are other inhibitory compounds in HEO_Fl_. Unfortunately, pure samples of the main compounds in HEO_Fl_, such as 3-methoxy-2,3-dimethylcyclobutene (MDCB, 9.8%), *cis*-*p*-menth-3-en-1,2-diol (9.1%), and 4-hydroxy-4-methyl-cyclohex-2-enone (HMCH, 3.4%), are not commercially available for testing. In HEO_Fl_, most of the unidentified compounds are oxygenated constituents, which we could not identify by MS data alone, and their relative amounts were <0.5% except for a few at ~1.0%. Since we identified 71.3% of the HEO_Fl_ components, the unidentified active components may also be present in the remaining 28.7% of unknown compounds, and further investigation will be needed to identify these components.

**Figure 3:**
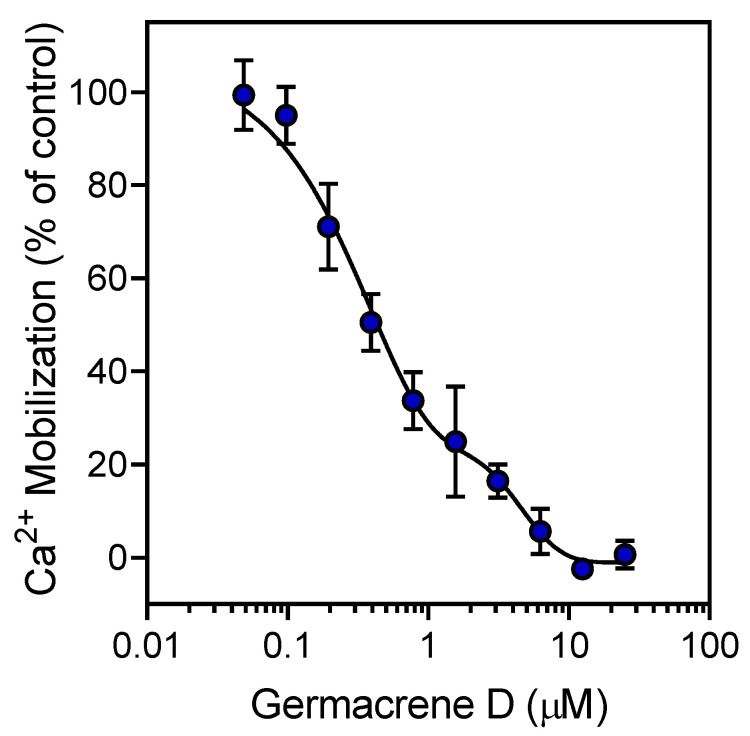
Inhibition of neutrophil Ca^2+^ mobilization by germacrene D. Human neutrophils were treated with the indicated concentrations of germacrene D or 1% DMSO (negative control) for 10 min. The cells were activated by 5 nM *f*MLF, and intracellular Ca^2+^ flux was monitored as described. The data are presented as the mean ± S.D. (N = 3) from one experiment that is representative of three independent experiments.

We also evaluated the effect of the sesquiterpenes germacrene D, α-humulene (also known as α-caryophyllene), and β-caryophyllene, which are principal components of HEO_Lv_, and the monoterpenoid geraniol, a minor component of both HEO_Fl_ and HEO_Lv_, on neutrophil Ca^2+^ mobilization induced by *f*MLF. As shown in Table 4, geraniol had no effect on *f*MLF-stimulated Ca^2+^ flux in human neutrophils. In contrast, all three sesquiterpenes potently inhibited *f*MLF-stimulated Ca^2+^ mobilization, with IC_50_ values in the sub-micromolar range. A representative concentration-dependent response for the inhibition of *f*MLF-induced neutrophil Ca^2+^ mobilization by germacrene D is shown in Figure 3.

**Figure 4:**
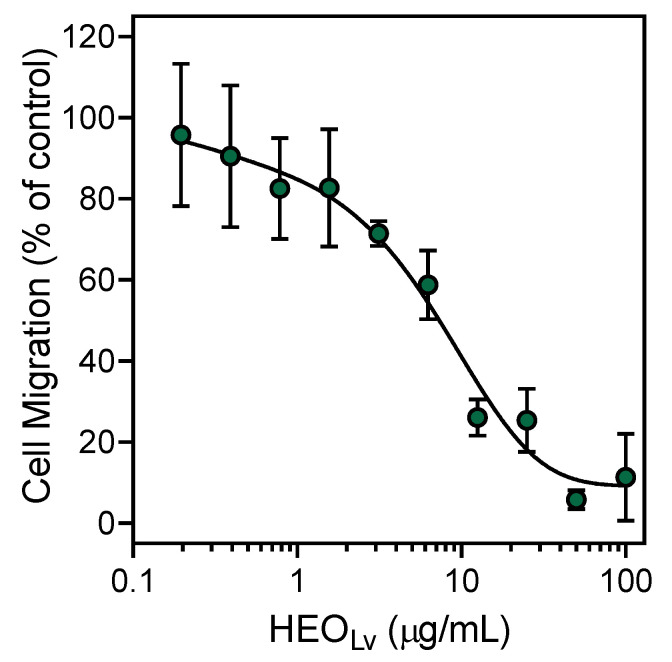
Inhibition of neutrophil chemotaxis by HEO_Lv_. Neutrophil migration toward 1 nM *f*MLF was measured, as described in Section 2. The data are presented as the mean ± S.D. (N = 3) from one experiment that is representative of two independent experiments.

**Figure 5:**
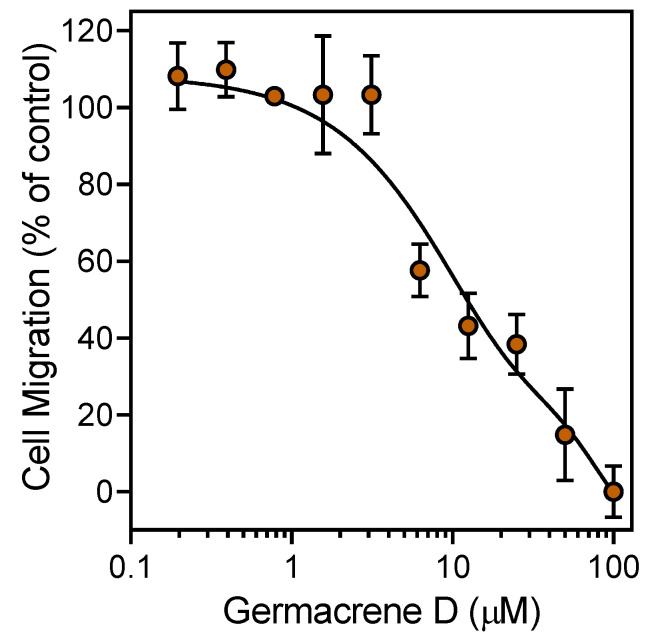
Inhibition of neutrophil chemotaxis by germacrene D. Neutrophil migration toward 1 nM *f*MLF was measured, as described in Section 2. The data are presented as the mean ± S.D. (N = 3) from one experiment that is representative of two independent experiments.

Various essential oils and their components have been reported previously to inhibit cell migration [24,50,51]. We found that pretreatment with HEO_Fl_ or HEO_Lv_ for 30 min concentration-dependently attenuated *f*MLF-induced neutrophil chemotaxis with IC_50_ values of 5.7 and 5.2 µg/mL, respectively (Table 4). In this case, the inhibitory effect of HEO_Lv_ was approximately the same as that of HEO_Fl_. A representative concentration-dependent response for the inhibition of neutrophil chemotaxis by HEO_Lv_ is shown in Figure 4. In our previous studies [24,26], we found that pretreatment with β-pinene, sabinene, and γ-terpinene inhibited neutrophil migration with IC_50_ values of 23.9, 39.1, and 32.5 µM, respectively. These active compounds compose 8.3% and 11.3% of HEO_Fl_ and HEO_Lv_, respectively. We also tested other commercially available components of the essential oils, including geraniol; myrtenol; terpinen-4-ol; α-terpineol; and the sesquiterpenes germacrene D, α-humulene, and β-caryophyllene, and found that only these three sesquiterpenes inhibited neutrophil migration, whereas the other compounds tested were inactive (Table 4). A representative concentration-dependent response for the inhibition of neutrophil chemotaxis by germacrene D is shown in Figure 5.

**Figure 6:**
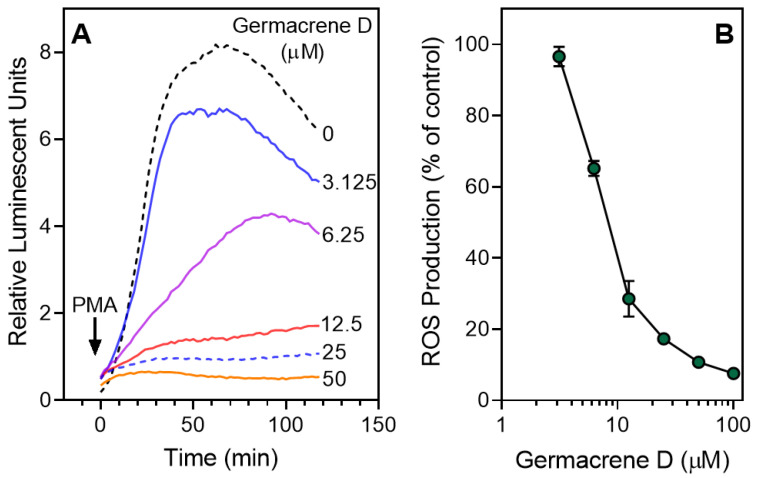
Inhibition of PMA-stimulated neutrophil ROS production by germacrene D. (**A**) Neutrophils were treated with 1% DMSO (negative control) or the indicated concentrations of germacrene D. After 10 min of preincubation, the cells were activated with 200 nM PMA (indicated by an arrow), and ROS production was monitored using an L-012-amplified assay system. (**B**) Relative integrated luminescence (120 min) is shown as the luminescence ratio normalized to background (1% DMSO) and plotted against germacrene D concentrations. The data are presented as the mean ± S.D. (N = 3) from one experiment. For both panels, a representative experiment from three independent experiments is shown.

Several essential oils have been reported to modulate ROS production by neutrophils [26,52,53]. Thus, we evaluated the effect of HEO_Fl_ and HEO_Lv_ on PMA-induced ROS production by human neutrophils and found that, similar to their effects on Ca^2+^ mobilization and chemotaxis, *Hypericum* essential oils inhibited ROS production, with HEO_Lv_ being ~8-fold more potent than HEO_Fl_. We also evaluated geraniol; myrtenol; terpinen-4-ol; α-terpineol; γ-terpinene; and the sesquiterpenes germacrene D, α-humulene, and β-caryophyllene in the same test-system and found that only the three sesquiterpenes inhibited ROS production in neutrophils, with IC_50_ values in the micromolar range (Table 3). As examples, representative concentration-dependent responses for inhibition of PMA-induced ROS production in human neutrophils treated by germacrene D are shown in Figure 6. Note also that none of the essential oils, monoterpenes, or sesquiterpenes that we evaluated directly activated ROS production by human neutrophils. Although various essential oils have been reported to modulate ROS production and Ca^2+^ mobilization in neutrophils [24,26,52,53], this is the first study to report the neutrophil immunomodulatory effects of essential oils isolated from *Hypericum* species, as well as the selected sesquiterpenes found in HEO_Lv_.

**Figure 7:**
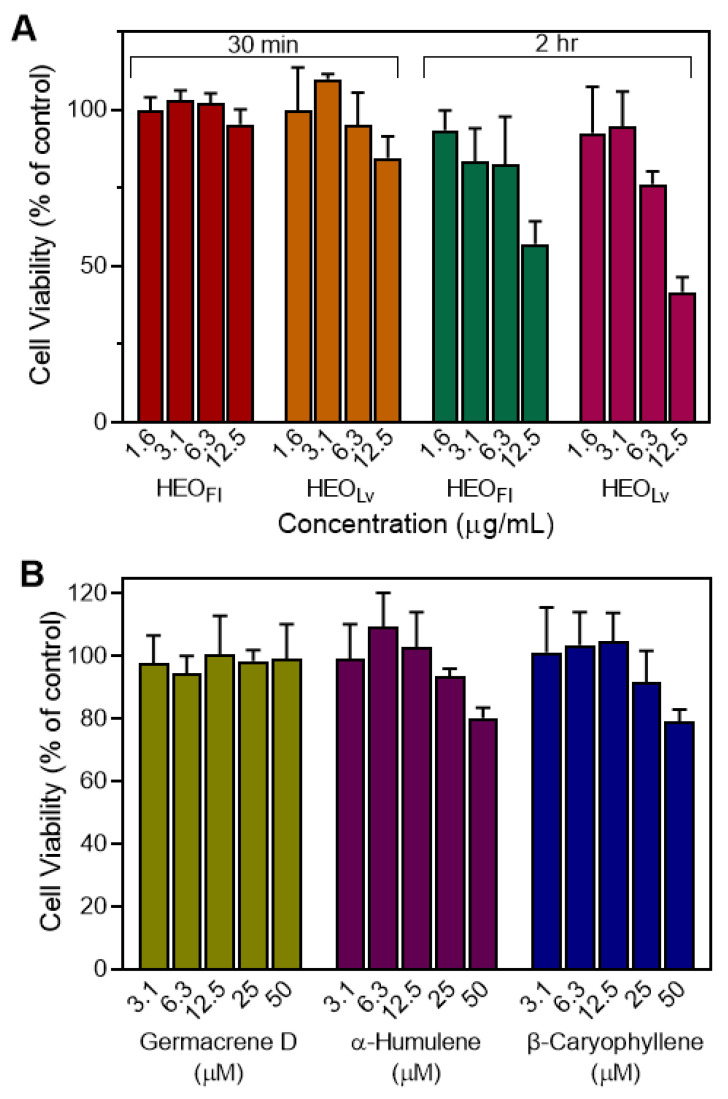
Cytotoxicity of HEO_Lv_, HEO_Fl_, and selected sesquiterpenes. HL-60 cells were preincubated with HEO_Lv_ or HEO_Fl_ for 30 min and 2 h (**A**) or with the indicated pure compounds for 2 h (**B**), and cell viability was analyzed as described. The data are presented as the mean ± S.D. (N = 3) from one experiment that is representative of two independent experiments.

To ensure that the results regarding inhibition of neutrophil functional activity (Ca^2+^ flux, cell migration, and ROS production) were not significantly influenced by potential toxicity, we evaluated cytotoxicity of HEO_Fl_, HEO_Lv_, germacrene D, α-humulene, and β-caryophyllene at various concentrations in HL-60 cells. As shown in Figure 7A, HEO_Fl_ and HEO_Lv_ had minimal cytotoxicity during a 30-min incubation. After 2 h, HEO_Fl_ and HEO_Lv_ exhibited some cytotoxic effects at 12.5 µg/mL. On the other hand, HEO_Lv_ demonstrated inhibitory activity in all three cell-based assays, and HEO_Fl_ inhibited chemotaxis assay at much lower concentrations (1–5 µg/mL, see Table 4). In addition, the inhibitory effects of HEO_Fl_ and HEO_Lv_ on neutrophil ROS production were observed during the first 30 min after PMA activation. Thus, it is unlikely that the inhibition of neutrophil responses by *Hypericum* essential oils was due to cytotoxicity at the concentrations and time periods tested. Furthermore, analysis of the pure sesquiterpenes showed that they had little or no cytotoxicity at all concentrations when tested over a 2 h incubation time (Figure 7B), again indicating that the inhibition of neutrophil responses by germacrene D, α-humulene, and β-caryophyllene was not due to cytotoxicity. 

Although some essential oils and their components were previously identified as HNE inhibitors [48,49], evaluation of HEO_Fl_; HEO_Lv_; and the sesquiterpenes germacrene D, α-humulene, and β-caryophyllene showed that they did not inhibit HNE, even at high tested concentrations (up to 50 μg/mL for the essential oils and 50 μM for the pure sesquiterpenes, data not shown). 

Clearly, HEO_Lv_ was most potent essential oil in our biological screening. Thus, we focused our biological evaluation on the major compounds in HEO_Lv_ that had not been investigated previously (i.e., the sesquiterpene compounds). Germacrene D, β-caryophyllene, α-humulene, and bicyclogermacrene are the main sesquiterpenes, representing 38.9% of HEO_Lv_. Although the germacrene D receptor was identified in neuronal cells of insects [54], biological activity of this compound in mammalian cells is completely unknown. In contrast, β-caryophyllene and α-humulene have been investigated in terms of their biological activity. For example, β-caryophyllene is a type 2 cannabinoid (CB2) receptor agonist and has been reported to inhibit α-glucosidase [55,56]. It also has anti-inflammatory activity in vitro and in vivo [57,58,59]. The reported immunomodulatory effects of β-caryophyllene include inhibition of microglial cells, CD4^+^ and CD8^+^ T lymphocytes, and expression of proinflammatory cytokines [59]. In addition, β-caryophyllene was reported to exert neuroprotective effects by modulating the expression of inflammatory mediators [60,61]. Furthermore, this sesquiterpene was reported to induce tumor cell apoptosis [62], prevent attachment of monocytic THP-1 cells to endothelial cells in vitro [63], and impair *Mycobacterium bovis* (BCG)-induced neutrophil accumulation in mouse pleurisy [64]. Although there are no direct data regarding inhibitory effects of this compound on ROS production, the caryophyllene-related sesquiterpenoids rumphellols A and B were reported to inhibit ROS production in human neutrophils [65].

α-Humulene is an isomer of β-caryophyllene, and these two compounds are often present as a mixture in some plants [66,67]. α-Humulene has been reported to inhibit nuclear factor (NF)-κB and activating protein (AP)-1 activity, the expression of P-selectin, and the increased mucus secretion in the lung in experimental airway allergic inflammation [68]. Both α-humulene and β-caryophyllene have been reported to inhibit lipopolysaccharide-induced NF-κB activation and neutrophil migration, although only α-humulene had the ability to prevent the production of the proinflammatory cytokines tumor necrosis factor (TNF) and interleukin (IL)-1β in a model of acute inflammation in rat paw [69]. α-Humulene and β-caryophyllene have also been reported to have acaricidal activities against *Dermatophagoides farinae* and *D. pteronyssinus* [70]. Finally, these sesquiterpenes inhibited cytochrome P4503A activity in rats and in human hepatic microsomes in vitro [71]. Thus, it is clear that the sesquiterpene compounds found in *H. perforatum* essential oils have a number of biological activities, including the neutrophil immunomodulatory activity reported here.

### 3.3. Identification of Potential Protein Targets for Selected Sesquiterpenes

Although we have not performed enantiomeric investigation of the main active sesquiterpenes of *H. perforatum* oils, (−)-β-caryophyllene was reported to be the most commonly found form of β-caryophyllene in many essential oils [72]. This enantiomeric form was also reported in essential oils of *Hypericum* species, including *H. perforatum* [73]. In nature, germacrene D occurs in two enantiomer forms, although the (−)-enantiomer is the most prevalent one found in higher plants [54,74]. Likewise, the (+)-configuration of bicyclogermacrene is the most common enantiomer in higher plants [75,76]. Thus, we performed reverse pharmacophore mapping on α-humulene and the enantiomeric structures of (−)-β-caryophyllene, (−)-germacrene D, and (+)-bicyclogermacrene to identify their potential biological targets. PharmMapper compared a large database of pharmacophore patterns with our test compounds and generated target information, including normalized fitness scores and pharmacophoric characteristics. 

As shown in Table 5, PharmMapper analysis indicated that among the eight top ranked potential targets, three targets were common for all sesquiterpenes and included serum albumin, aldo-keto reductase family 1 member C2 (AKR1C2), and mitogen-activated protein kinase (MAPK)-activated protein kinase 2 (MAPKAPK2 or MK2). Bone morphogenetic protein 2 (BMP-2) was a common target for (−)-β-caryophyllene, α-humulene, and bicyclogermacrene. Apolipoprotein A-II (ApoA-II) and kinesin-like protein KIF11 were potential targets for (−)-β-caryophyllene, (−)-germacrene D, and bicyclogermacrene. Steroid sulfatase was a target for (−)-β-caryophyllene, (−)-germacrene D, and α-humulene. Proviral integration Moloney virus kinase (PIM1) and caspase-7 were potential targets for (−)-germacrene D, α-humulene, and bicyclogermacrene. Integrin α-L (CD11a) was a target for (−)-β-caryophyllene, and thyroid hormone receptor β (THRB) was a potential target for α-humulene only. 

Among these potential protein targets, only CD11a, MAPKAPK2, and PIM1 could explain the direct inhibitory effect of HEO_Lv_ and its primary sesquiterpenes on human neutrophil functional activities, including inhibition of ROS production and chemotaxis. Indeed, neutrophil arrest and migration involves integrin α-L (CD11a) [77]. Upon activation of p38 MAPK, MAPKAPK2 binds to p38 MAPK, leading to phosphorylation of Hsp27, Akt, and Cdc25, which are involved in regulation of various essential cellular functions [78]. In support of this idea, MAPKAPK2^−/−^ neutrophils generated less O_2_^−^, and both NADPH-oxidase activation and p47^phox^ phosphorylation were decreased [79]. PIM kinases have been reported to promote cell migration and invasion [80], and participation of PIM1 in p22^phox^-dependent signaling also was reported [81]. 

Since MAPKAPK2 and PIM1 could interfere with NADPH-oxidase activation and suppress phagocyte migration and ROS production, we evaluated the binding affinity of pure β-caryophyllene, α-humulene, and germacrene D toward these two kinases using KINOMEscan but did not find any binding activity. 

It should be noted that β-caryophyllene oxide was completely inactive in human neutrophils. We also conducted PharmMapper analysis for this compound and found that CD11a (fit score = 0.998) was its best ranked potential protein target. Other potential protein targets for β-caryophyllene oxide are KIF11 (fit score = 0.980), AKR1C2 (0.979), BMP-2 (0.966), MAPKAPK2 (0.965), steroid sulfatase (0.936), caspase-7 (0.930), and PIM1 (0.923). Thus, because CD11a is a potential target for both β-caryophyllene oxide and β-caryophyllene, this protein is unlikely to be a relevant target for β-caryophyllene in human neutrophils. 

Using the SwissADME online tool [31], we calculated the most important physicochemical parameters for the sesquiterpenes, including β-caryophyllene oxide (Table 6), and found that the compounds are very similar to each other in terms of many ADME properties. Nevertheless, they differed noticeably in topological polar surface area (tPSA) and Log P. These descriptors are usually related to the capacity of molecules to cross cellular membranes [82]. For example, it was reported earlier that compounds with LogP > 4 and TPSA < 40 Å^2^ had optimal antimycobacterial activity [83]. Thus, it is possible that the inactivity of β-caryophyllene oxide in human neutrophils could be explained by low cell membrane permeability, as this compound is less lipophilic and more polar than the investigated sesquiterpenes of purely hydrocarbon nature.

## 4. Conclusions

We report here that essential oils isolated from leaves of *H. perforatum* contain a high amount of sesquiterpenes and that these essential oils are potent inhibitors of human neutrophil functional responses. Moreover, the essential oil constituents germacrene D, β-caryophyllene, and α-humulene were also potent inhibitors of *f*MLF-induced Ca^2+^ mobilization, chemotaxis, and ROS production by human neutrophils. Thus, our data provide a molecular basis to explain at least part of the beneficial therapeutic effects of essential oils from *H. perforatum* and suggest that suppression of neutrophils by the essential oil components of this plant might have anti-inflammatory effects. Future studies are now in progress to evaluate the potential of *Hypericum* essential oils as therapeutic remedies for various disorders with immune and/or inflammatory mechanisms, as well as to determine molecular targets of their active components.

## Figures and Tables

**Table 1:** Review of the major volatile constituents of *H. perforatum* essential oils (2010–2020).

Location	Major Compound (%)	Ref.
Greece	α-pinene (7.5); (*E*)-β-caryophyllene (10.3); germacrene D (5.5); β-selinene (14.7); α-selinene (14.6)	[39]
Iran	decane (27.7); menthol (8.9); methyl decanoate (4.6); β-elemene (4.6)	[37]
	eudesma-4(15),7-dien-1β-ol (8.1–7.5); thymol (7.0–7.2); 1,4-*trans*-1,7-*trans*-acorenone (5.2–5.5)	[37]
	decane (59.6); dodecane (12.9); ethyl cyclohexane (6.8); 5-methyl nonane (4.7); 3-methyl nonane (4.3)	[36]
	2,6-dimethyl-heptane (6.2–36.1); α-pinene (5.6–26.0); δ-cadinene (0.0–22.6); γ-cadinene (0.0–16.9)	[42]
	α-pinene (12.5); β-pinene (8.3); (*E*)-β-ocimene (4.4); 2-methyl decane (4.0); undecane (7.0); germacrene D (6.9); α-selinene (4.2)	[33]
	*α*-pinene (21.9); nonane (9.8); *n*-octane (9.1); dodecanol (6.8)	[43]
Serbia	germacrene D (18.6); β-caryophyllene (11.2); 2-methyl octane (9.5); α-pinene (6.5); bicyclogermacrene (5.0); (*E*)-β-ocimene (4.6)	[34]
Syria	β-selinenol (18.1); elemol (12.8); β-elemene (10.7)	[38]
Turkey	β-selinene (19.4); bicyclogermacrene (15.3); tetradecene (8.2); α-amorphene (8.1)	[35]

**Table 2:** Chemical composition of essential oils obtained from *H. perforatum* flowers (HEO_Fl_) and leaves (HEO_Lv_) ^a^.

N^o^	RRI	Compound	Fl	Lv	N^o^	RRI	Compound	Fl	Lv
1	965	3-methyl nonane		0.6	48	1687	α-humulene		1.2
2	1032	α-pinene	2.2	4.9	49	1690	cryptone	0.9	
3	1035	α-thujene		1.4	50	1693	β-acoradiene		0.4
4	1048	MBOL	1.5		51	1700	limonen-4-ol	3.2	
5	1065	2-methyl decane		0.2	52	1704	γ-muurolene		1.4
6	1100	undecane		0.2	53	1706	α-terpineol	6.1	
7	1118	β-pinene	0.9	4.6	54	1708	ledene		0.3
8	1132	sabinene		5.6	55	1726	germacrene D		25.7
9	1174	myrcene		2.1	56	1740	α-muurolene		0.8
10	1176	α-phellandrene		t	57	1743	α-cadinene		0.3
11	1188	α-terpinene		0.5	58	1743	eremophilene		1.7
12	1203	limonene		0.5	59	1755	bicyclogermacrene		2.5
13	1218	β-phellandrene		1	60	1758	(*E*,*E*)-α-farnesene		0.7
14	1225	(*Z*)-3-hexenal		0.2	61	1773	δ-cadinene		2.5
15	1246	(*Z*)-β-ocimene		0.5	62	1776	γ-cadinene		1.0
16	1255	γ-terpinene		1.1	63	1799	cubenene		0.1
17	1266	(*E*)-β-ocimene		4.2	64	1804	myrtenol	2.7	
18	1280	*p*-cymene	0.5	1.2	65	1815	2,6-dimethyl-3(*E*),5(*Z*),7-octatriene-2-ol		0.1
19	1290	terpinolene		0.3	66	1830		1.5	
20	1466	α-cubebene		0.1	67	1853	*cis*-calamenene		0.2
21	1475	acetic acid	1.3		68	1857	geraniol	1.4	t
22	1493	α-ylangene		0.1	69	1864	*p*-cymen-8-ol	2.9	0.1
23	1495	bicycloelemene		0.1	70	1900	*epi*-cubebol		t
24	1497	α-copaene		0.2	71	1945	1,5-epoxy-salvial(4)14-ene		t
25	1506	decanal		t	72	1953	palustrol		0.4
26	1525	α-funebrene		t	73	1973	dodecanol		1.2
27	1535	β-bourbonene		0.2	74	2006	MDCB *	9.8	
28	1536	italicene		t	75	2008	caryophyllene oxide		1.2
29	1544	α-gurjunene		0.5	76	2050	(*E*)-nerolidol		0.8
30	1549	β-cubebene		t	77	2057	ledol		1.2
31	1553	linalool	1.3	0.2	78	2069	germacrene D-4β-ol		t
32	1562	octanol	1.1		79	2080	cubenol		0.2
33	1571	TMEOL	0.9	t	80	2088	1-*epi*-cubenol		0.2
34	1586	pinocarvone	0.9		81	2093	junenol		0.3
35	1587	β-funebrene		1.1	82	2098	globulol		0.3
36	1589	β-ylangene		0.3	83	2099	*trans*-ascaridol glycol *	4.6	
37	1602	MHDO	t		84	2104	viridiflorol		0.2
38	1608	β-pinone	1.3		85	2115	HMCH	3.4	
39	1608	β-copaene		0.4	86	2144	spathulenol	1.6	1.9
40	1611	terpinen-4-ol	7.4	2.6	87	2179	tetradecanol		0.7
41	1612	β-caryophyllene	t	9.5	88	2184	*cis*-*p*-menth-3-en-1,2-diol	9.1	
42	1613	β-cedrene		0.4	89	2187	T-cadinol		0.6
43	1638	CMEOL	1.1		90	2209	T-muurolol		0.8
44	1661	alloaromadendrene		0.6	91	2219	torreyol		0.2
45	1668	(*Z*)-β-farnesene		0.6	92	2255	α-cadinol	1.5	1.9
46	1670	*trans*-pinocarveol	0.6		93	2260	alismol		0.1
47	1683	*trans*-verbenol	1.6		94	2329	*trans*-sobrerol *	1.7	

^a^ The data are presented as relative % by weight for each component isolated from *H. perforatum* flowers and leaves. RRI was calculated based on retention of n-alkanes; %, calculated from flame ionization detector data. Trace amounts (t) were present at <0.1%. * Tentatively identified using Wiley and MassFinder mass spectra libraries and published RRI. All other compounds were identified by comparison with co-injected standards. Abbreviations: CMEOL, *cis*-*p*-menth-2-en-1-ol; HMCH, 4-hydroxy-4-methyl-cyclohex-2-enone; MBOL, 2-methyl-3-buten-2-ol; MDCB, 3-methoxy-2,3-dimethylcyclobutene; MHDO, 6-methyl-3,5-heptadien-2-one; RRI, relative retention index; TMEOL, *trans*-*p*-menth-2-en-1-ol.

**Table 3:** Summary of the chemical compositions of HEO_Fl_ and HEO_Lv_.

Total (%)	71.3	97.2
Monoterpene hydrocarbons (%)	3.6	27.9
Oxygenated monoterpenes (%)	49.2	3.0
Sesquiterpene hydrocarbons (%)	0	52.9
Oxygenated sesquiterpenes (%)	3.1	10.3
Miscellaneous compounds (%)	15.4	3.1

**Table 4:** Biological activity of HEO_Fl_ and HEO_Lv_ and their commercially available constituent compounds in human neutrophils.

Essential Oil or Pure Compound	Composition (%)		Ca^2+^ Flux ^a^	Chemotaxis ^b^	ROS Production ^c^
			IC_50_ (μg/mL)		
HEO_Fl_			11.3 ± 1.8	5.7 ± 1.8	9.5 ± 0.9
HEO_Lv_			2.3 ± 0.4	5.2 ± 1.1	1.2 ± 0.4
	HEO_Fl_	HEO_Lv_	IC_50_ (μM)		
Caryophyllene oxide	0	1.2	N.A. ^d^	N.A. ^d^	N.A. ^d^
*p*-Cymen-8-ol	2.9	0.1	N.A. ^d^	N.A. ^d^	N.A. ^d^
*p*-Cymene	0.5	1.2	N.A. ^d^	N.A. ^d^	N.A. ^d^
Myrcene	0	2.1	N.A. ^d^	N.A. ^d^	N.A. ^d^
α-Terpinene	0	0.5	N.A. ^d^	N.A. ^d^	N.A. ^d^
Limonene	0	0.5	N.A. ^d^	N.A. ^d^	N.A. ^d^
Myrtenol	2.7	0	N.A. ^d^	N.A.	N.A.
(*E/Z*)-β-Ocimene	0	4.7	N.A. ^d^	N.A. ^d^	N.A. ^d^
α-Pinene	2.2	4.9	N.A. ^d^	N.A. ^d^	N.A. ^d^
(1*S*)-(-)-β-Pinene	0.9	4.6	N.A. ^d^	22.7 ± 2.6 ^d^	N.A. ^d^
(±)-Sabinene	0	5.6	N.A. ^d^	37.4 ± 4.3 ^d^	N.A. ^d^
(−)-Terpinen-4-ol	7.4	2.6	N.A. ^d^	N.A.	N.A.
γ-Terpinene	0	1.1	N.A.	32.5 ± 4.6 ^d^	N.A.
α-Terpineol	6.1	0	N.A. ^d^	N.A.	N.A.
Terpinolene	0	0.3	N.A. ^d^	N.A. ^d^	N.A. ^d^
(-)-Linalool	1.3	0.2	N.A. ^d^	N.A. ^d^	N.A. ^d^
MHDO	< 0.1	0	8.2 ± 2.5 ^d^	3.6 ± 0.5 ^d^	2.8 ± 0.4 ^d^
Germacrene D	0	25.7	0.51 ± 0.08	5.4 ± 2.3	9.9 ± 1.9
Geraniol	1.4	< 0.1	N.A.	N.A.	50.1 ± 3.2
α-Humulene	0	1.2	0.31 ± 0.06	12.0 ± 3.4	2.2 ± 0.8
β-Caryophyllene	< 0.1	9.5	0.33 ± 0.02	17.6 ± 5.7	2.6 ± 0.9

^a^ Inhibition of neutrophil Ca^2+^ flux induced by 5 nM *f*MLF. ^b^ Inhibition of neutrophil chemotaxis toward 0.5 nM *f*MLF. ^c^ Inhibition of neutrophil ROS production induced by 200 nM PMA. N.A.: no activity was observed, even at the highest concentration tested (50 µM). ^d^ Previously reported data [48,49]. IC_50_ values are presented as the mean ± S.D. of three independent experiments, as described in Section 2.

**Table 5:** Identification of potential protein targets of (−)-β-caryophyllene, (−)-germacrene D, α-humulene, and bicyclogermacrene.

Rank	PDB ID	Target Name	Fit Score	Rank	PDB ID	Target Name	Fit Score
(−)-β-Caryophyllene				α-Humulene			
1	1XDD	Integrin α-L	0.998	1	2P3G	MAPKAPK2	0.989
2	1REU	BMP-2	0.987	2	3BMP	BMP-2	0.987
3	2P3G	MAPKAPK2	0.981	3	1J96	AKR1C2	0.981
4	1J96	AKR1C2	0.981	4	1SHJ	Caspase-7	0.961
5	1L6L	ApoA-II	0.957	5	2O65	PIM1	0.925
6	1P49	Steroid sulfatase	0.954	6	1P49	Steroid sulfatase	0.908
7	1E7E	Serum albumin	0.919	7	2PIN	THRB	0.885
8	2PG2	KIF11	0.919	8	1E7A	Serum albumin	0.880
**(** **−)-Germacrene D**				**Bicyclogermacrene**			
1	1E7A	Serum albumin	0.999	1	1REU	BMP-2	0.998
2	1SHJ	Caspase-7	0.990	2	2O65	PIM1	0.993
3	1P49	Steroid sulfatase	0.977	3	1E7E	Serum albumin	0.991
4	2O65	PIM1	0.969	4	1J96	AKR1C2	0.990
5	2PG2	KIF11	0.958	5	2P3G	MAPKAPK2	0.988
6	1L6L	ApoA-II	0.951	6	2PG2	KIF11	0.920
7	2P3G	MAPKAPK2	0.948	7	1L6L	ApoA-II	0.913
8	1J96	AKR1C2	0.924	8	1SHJ	Caspase-7	0.913

Abbreviations: AKR1C2, aldo-keto reductase family 1 member C2; BMP-2, bone morphogenetic protein 2; KIF11, kinesin-like protein KIF11; MAPKAPK2, mitogen-activated protein kinase-activated protein kinase 2; THRB, thyroid hormone receptor β; ApoA-II, apolipoprotein A-II; PDB ID, the 4-character unique identifier of every entry in the Protein Data Bank.

**Table 6:** Physicochemical properties of (−)-germacrene D, bicyclogermacrene, α-humulene, (−)-β-caryophyllene, and β-caryophyllene oxide.

Property	Germacrene D	Bicyclo- germacrene	α-Humulene	β-Caryophyllene	β-Caryophyllene Oxide
Formula	C_15_H_24_	C_15_H_24_	C_15_H_24_	C_15_H_24_	C_15_H_24_O
M.W.	204.35	204.35	204.35	204.35	220.35
Heavy atoms	15	15	15	15	16
Fraction Csp^3^	0.60	0.73	0.60	0.73	0.87
Rotatable bonds	1	0	0	0	0
H-bond acceptors	0	0	0	0	1
H-bond donors	0	0	0	0	0
MR	70.68	68.78	70.42	68.78	68.27
tPSA	0.00	0.00	0.00	0.00	12.53
Log P	4.30	4.15	4.26	4.24	3.68

Abbreviations: M.W., molecular weight (g/mol); MR, molar refractivity; tPSA, topological polar surface area (Å^2^); Log P, lipophilicity (consensus Log Po/w).
